# The Medical Schools

**Published:** 1901-09-07

**Authors:** 


### MEDICAL QUALIFICATIONS.

THE following is a complete list of the bodies granting full qualifications, which are accepted by the General Medical Council as admitting to the Register:—

THE UNIVERSITY OF OXFORD.

THE UNIVERSITY OF CAMBRIDGE.

THE UNIVERSITY OF LONDON.

THE UNIVERSITY OF DURHAM.

THE VICTORIA UNIVERSITY.

THE UNIVERSITY OF BIRMINGHAM.

THE UNIVERSITY OF EDINBURGH.

THE UNIVERSITY OF GLASGOW.

THE UNIVERSITY OF ABERDEEN.

THE UNIVERSITY OF ST. ANDREWS.

THE UNIVERSITY OF DUBLIN.

THE ROYAL UNIVERSITY OF IRELAND.

THE SOCIETY OF APOTHECARIES OF LONDON.

### THE MEDICAL SCHOOLS.

FOR the guidance of those about to enter we give the following complete list of the medical schools in the United Kingdom. So far as the English schools are concerned, we give the number of beds in the hospitals to which they are attached; but too much stress must not be laid upon mere size, some of the schools attached to small hospitals being admirable in all that concerns teaching. Still, other things being equal, there are certain advantages in having the run of a large hospital, and we are distinctly of opinion that it is an advantage to belong to a fairly large school, one having a considerable number of students. At such a place things are apt to be better done, and the student is more sure of finding classes ready for him on whatever subject he may be working, than where the numbers are smaller.

Under the head "composition fee" we have given the sum charged by each hospital and school for all the classes, lectures, and hospital attendance which are required for the diplomas of the Conjoint Examining Board, but at all the schools special arrangements will be made to suit special requirements.

For further particulars application should be made to the particular schools or hospitals concerning which information is desired, the person to address being usually "The Dean of the Medical School."

At some of the hospitals, although students are accepted for short periods, only full term pupils are eligible for the house appointments. This must be borne in mind and inquired about by those students who, after pursuing the earlier portion of their course at Cambridge or other such place, wish to do their hospital practice in London, for the higher hospital appointments are the great prizes.

### LONDON MEDICAL SCHOOLS.

### ST. BARTHOLOMEW'S HOSPITAL MEDICAL SCHOOL,
*West Smithfield, E.C.*

St. Bartholomew's is one of the largest hospitals, and the school attached to it may be regarded as the most important of all the medical schools in London.

The hospital was founded in the year 1123 by Rahere, who subsequently founded the Priory of St. Bartholomew. From the beginning it was a hospital for the sick and not a mere almshouse. This is distinctly expressed in a grant of privileges to it by Edward III. The earliest record of the school dates from 1662. Many additions were made from time to time, and in 1876 the old buildings were removed and replaced on a larger area. This design was completed in 1881 at a cost of £50,000. In 1890 a bacteriological laboratory was added, and in 1891 new laboratories for public health and for biology were built.

A complete convalescent hospital at Swanley, in Kent, forms an important part of St. Bartholomew's Hospital.

The clinical practice of the hospital now comprises a service of 744 beds including the 70 at Swanley.

The total value of the scholarships and prizes is nearly £900 per annum.

There is a resident college for students within the precintcs of the hospital, and a very admirable recreation ground has been acquired at Winchmore Hill, which is easily reached by train.

The composition fee if paid in one sum is 150 guineas, and special arrangements are made for those who have already completed their anatomical and physiological studies.

### GUY'S HOSPITAL MEDICAL SCHOOL,
*London Bridge, S.E.*

Guy's Hospital contains more than 600 beds, of which 550 are in constant occupation. Thirty-six beds are set apart for diseases of the eye, and 40 for a selection of the most urgent and interesting medical cases which form the subjects of the clinical lectures. This collection of interesting cases in the clinical ward is a matter of great convenience and advantage to the students.

A large ward which has been closed for fifteen years has now been opened for the reception of diseases of women, and a few beds are reserved for cases of difficult labour—again a matter of much advantage for those studying midwifery. There are many scholarships in connection with the school, and the appointments in the hospital are numerous and all free. Rooms and commons are provided for all who hold "indoor" appointments, and also for all dressers during their "take-in" week.

The special departments at Guy's are well developed, and there is a particularly flourishing school of dental surgery.

The Clubs' Union recreation ground at Honor Oak Park is easily reached from the hospital.

The composition fee is 150 guineas.

St. Thomas's Hospital Medical School,
*Albert Embankment, S.E.*

The present hospital contains in all 572 beds, of which about 512 are in use. From its foundation down to the year 1862, the hospital occupied the original site where it had stood since the year 1228, when the then new building replaced what was then "an ancient hospital built of old" which had been destroyed by fire 21 years previously. In 1862, however, the site was sold for the extension of London Bridge Station, and in 1871 the new building was opened by her Majesty Queen Victoria. The hospital, as it stands at present, is a modern building on the pavilion plan, occupying a splendid position opposite the Houses of Parliament, and so near to Waterloo Station that junior students can with the greatest convenience live a little way out of town, which is often a great advantage. Considerable alterations have been an d are still going on with the object of providing better accommodation in the operation rooms and separate wards for children.

The school buildings are well adapted for their purpose. There is a good club attached at which students can obtain their meals, so that they can spend the whole day at the school, and a good recreation ground with pavilion is provided at Chiswick.

There are a considerable number of scholarships, and all the hospital appointments are open to students without charge. The composition fee is £150.

King's College Medical Faculty,
*Strand, W.C.*

King's College Hospital is situated in Portugal Street, Lincoln's Inn, W.C., about five minutes' walk from the College. King's and University are two completely equipped colleges, the educational facilities offered by which extend far beyond medicine. Indeed, the Medical is but one of many Faculties. Thus the medical students at both King's College and at University College form but a part of a large mass of students who are working at a very considerable range of subjects. Theoretically, one would say that this would be a great advantage, and that the intermingling of those studying arts, sciences, and literature ought to have an important influence upon them by eliminating that narrowness of view which is so apt to characterise those who specialise their studies early in life. We are afraid, however, that at both these colleges the medical students tend very much to keep together and thus to lose one of the great advantages offered to them by these institutions.

King's College Hospital is not very large, the number of beds being only 220, but it is very well arranged for teaching purposes, and a considerable number of appointments are open to the students. At the college also there are a considerable number of scholarships. It may be mentioned that the college is more particularly noted for the completeness of the opportunities afforded for the study of State Medicine.

The composition fee is £135 if paid in one sum.

University College, London, Medical Faculty,
*Gower Street, W.C.*

The hospital contains 185 beds, but the new buildings, one half of which are now completed and in use, will probably increase this number. The new hospital which is being erected by Sir J. Blundell Maple is admirably arranged from the students' point of view, the shortness of the corridors between the wards being a cause of much saving of time, and all the laboratory arrangements for the scientific investigation of the cases being most complete and convenient. University College, where the study of the ancillary sciences is undertaken, faces the hospital on the other side of Gower Street. This is an institution of the same type as King's College, although even more complete and wider in its range of teaching. There are several entrance and other scholarships, prizes to the annual amount of about £500 being given by the Faculty of Medicine, and all the appointments in the hospital are open to the students without extra fee.

The composition fee is 150 guineas.

Charing Cross Hospital Medical School,
*Charing Cross, W.C.*

This hospital occupies a remarkably advantageous position in the very centre of London, and its students enjoy exceptional opportunities, a large number of street accidents and emergency cases of all kinds being brought in.

The hospital contains 180 beds, and the additions now being made will increase the accommodation by 70 beds, in addition to which the existing buildings are being entirely remodelled.

For a hospital of its size there are a considerable number of house appointments, and as from its position it is of necessity kept constantly in full swing it offers a great field for the student.

There are five Entrance Scholarships open to students commencing their medical education, three of which (100 guineas, 60 guineas, and 40 guineas) are open to all general students, one is reserved for the sons of medical men, and one is open to dental students only.

There are several entrance scholarships, University Scholarships open to students of a certain standing at Oxford and Cambridge; and an Epsom Scholarship is each winter placed at the disposal of the Royal Benevolent College to be competed for by their foundation scholars who have passed the Preliminary Scientific M.B. Examination of the University of London.

A considerable number of scholarships and prizes are open to the students of the school during their curriculum. The composition fee is 110 guinees.

St. George's Hospital Medical School,
*Hyde Park Corner, S.W.*

This hospital occupies an admirable site at Hyde Park Corner, and besides serving the district in its immediate neighbourhood is the principal hospital for that large westward extension of London which has grown up during the past few years, a large proportion of which consists of houses of a more or less working-class character. The hospital contains 351 beds. There are several entrance and other scholarships, one of which is restricted to the sons of medical men, and one open only to sons of officers in His Majesty's service who have met their death in the South African War. Two scholarships are open only to those who have passed or completed the curriculum for the Oxford First M.B. or the Cambridge Second M.B., and another for students of Provincial University Colleges who have completed the first half of their curriculum. The composition fee is £150.

LONDON HOSPITAL MEDICAL SCHOOL,

*Mile End, E.*

This hospital is situated in the East End of London in the midst of a vast labouring population. There are docks and factories on every side, from which and from the crowded streets there pours in an endless stream of accidents and casualties of all kinds. This is indeed the great accident hospital of London, and from the enormous number of patients who flock to its doors it affords an almost unique field for the study of disease. It is the largest general hospital in the kingdom, and contains nearly 800 beds.

All the special departments are very well provided for, and lately the hospital has obtained considerable fame by the publicity given to its arrangements for the special treatment of lupus in accordance with the plan of Professor Finsen, of Copenhagen. A large number of scholarships and prizes are open to the students, and, as one would expect in such a large hospital, the list of appointments is almost endless. These are open to the students, and from the many opportunities which they offer to them for taking personal and responsible charge of patients, under supervision, they are of great value. Many of these appointments are salaried.

The new departments of Bacteriology, Public Health, Chemistry, and Biology, and the new Pathological Institute at the hospital are now completed and in full use. During the last year new students' rooms have been added, and a garden and Fives Court opened for the use of the Clubs' Union.

There is also an athletic ground within easy reach, and from the immediate propinquity of the various railway stations it is easy for the students to find lodgings in pleasant suburbs.

The composition fee is 120 guineas, a reduction of 15 per cent. being made to sons of medical men.

ST. MARY'S HOSPITAL MEDICAL SCHOOL,
*Paddington, W.*

The hospital at the present time contains 281 beds, 128 of which are devoted to medical and 153 to surgical cases. The Clarence Wing, the ground floor of which was opened in 1898, is about to be completed, and when this is done it will increase the number of beds to about 380. The out-patient department is very large, and offers an unlimited field for observing all varieties of disease. The situation of the hospital in the midst of a pleasant residential quarter offers great advantages to students who require to live near their work, while the railway, which is only just over the way, renders access to the country and the river peculiarly easy. The number of operations performed has greatly increased during recent years, and the principal operating theatre has been enlarged and entirely reconstructed. All clinical appointments as clerks and dressers are of four months' duration, so that the students have the advantage of a somewhat longer experience of this work than is demanded by the Examining Boards. All the clinical appointments are free, and the residents, who are chosen by competitive examination, receive board and residence in the hospital. There are several good entrance scholarships, and with a view to encouraging students to enter for the higher examinations all the special classes held for those so doing are thrown open to all full students without any additional fees.

The composition fee is £140 if paid in one sum, and includes membership of the amalgamated clubs. Special fees are arranged for those students who have completed their examinations in anatomy and physiology at Oxford, Cambridge, or other British Universities.

MIDDLESEX HOSPITAL MEDICAL SCHOOL,
*Berners Street, W.*

The hospital contains 340 beds. The study of cancer has for long been a special feature at this hospital, and a new wing containing 40 beds, and special research laboratories are now entirely devoted to patients suffering from the disease. Thus the Middlesex Hospital offers unrivalled opportunities for the study of this disease both in its clinical and its pathological aspects.

There are several entrance scholarships, and eighteen resident appointments are open annually to competition among pupils of the hospital. The officers reside in the residential college free of expense. This residential college is attached to the hospital and overlooks the hospital grounds. It accommodates about 30 students. The non-residential appointments also are numerous, and they are so arranged that each student may, during his course, hold all the out-patient and in-patient clerkships and dresserships.

The entrance fee is 135 guineas.

WESTMINSTER HOSPITAL MEDICAL SCHOOL,
*Caxton Street, S.W.*

The Westminster Hospital stands just opposite Westminster Abbey, and the school is close by in Caxton Street.

The hospital contains 212 beds. A medical and a surgical registrar are appointed annually, each with a salary of £40.

Two house-physicians, two house-surgeons, two assistant house-surgeons, and a resident obstetric assistant, are appointed for six months and are provided with rooms and commons in the hospital, except the assistant house-surgeons, who have commons only.

The students' appointments as clerks and dressers are held for four months each.

This is one of the smaller schools, but for its size it is peculiarly well endowed with scholarships, among which is one offering a free medical education to pupils of Epsom College. If we may judge from the prospectus a more earnest endeavour seems to be made at this school than at some others to keep the students to their work.

The composition fee is 110 guineas, which includes the subscription to the Clubs' Union for five years.

LONDON (ROYAL FREE HOSPITAL) SCHOOL OF MEDICINE FOR WOMEN,
*30 Handel Street, Brunswick Square, W.*

The Royal Free Hospital, in connection with which the London School of Medicine for Women is conducted, contains 170 beds, all of which are available for clinical instruction. There are separate departments for the various specialties. Students of the school also attend the practice of one of the Fever Hospitals of the Metropolitan Asylums Board, and receive instruction in lunacy at one of the asylums of the London County Council. Arrangements have also been made for the students of this school to attend the practice of a number of these specials.

There are various entrances and other scholarships in connection with the school; the usual dresserships and clerkships at the hospital open to the students, and after qualification various resident and other appointments are available.

In addition to the ordinary scholarships, various Missionary Societies also offer scholarships on certain conditions, and assist ladies who wish to go to India as medical missionaries.

The school has been recently rebuilt and much enlarged. The laboratories are large, well lighted, and fully equipped. There is a large Library and Common Room, and there are sets of chambers to accommodate seventeen students.

The composition fee is £125.

### Cooke's Medical School, London.

This is a school chiefly engaged in the special preparation of students for particular examinations, and is devoted principally to the teaching of anatomy, physiology, and operative surgery on the dead body. Its courses are recognised for Army promotions, and for the Indian Medical Service, and by decision of the various Examining Bodies students rejected at their examinations in anatomy and physiology can get "signed up" from this school for the three months' supplementary work which is required of them before submitting themselves for re-examination. The school is also recognised for the special dissections required for the Fellowship of the Royal College of Surgeons.

---

### PROVINCIAL MEDICAL SCHOOLS.

No one need nowadays be ashamed of being a provincial student. The public position occupied by the provincial schools, the admirable hospitals in which the medical instruction is given, and the advanced position occupied by the leading provincial teachers and practitioners, offer to the student everything that is required for obtaining a thoroughly sound professional education, while many of the provincial schools give him far greater opportunities than he has in London of obtaining that public recognition of his work which is expressed by a University degree. The medical schools in England, outside the metropolis, in which a complete medical education can be obtained are the Universities of Oxford, Cambridge, and Durham (University of Durham College of Medicine, Newcastle-on-Tyne); the three colleges of the Victoria University (Owens College, Manchester; University College, Liverpool; and the Yorkshire College, Leeds); the University of Birmingham (Mason University College, Birmingham); University College, Bristol; and University College School of Medicine, Sheffield.

It will be noted that most of these are in direct connection with their own Universities, but at all of them alike it is possible to prepare as external students for the medical degrees of the University of London, or for any of the College and other diplomas.

In addition to these we must mention the Medical School of University College of South Wales at Cardiff, which gives instruction in all the subjects included in the first three years of the medical curriculum.

### Oxford and Cambridge.

These are mostly regarded as places for the study of the preliminary sciences and of anatomy and physiology. For this they offer the very highest facilities. In regard to hospital work instruction in clinical medicine, surgery and midwifery is given by University lecturers and by the visiting staffs of the Radcliffe Infirmary at Oxford and of Addenbrook's Hospital at Cambridge; but the usual practice is for men, after spending three years at the University and passing their examinations in anatomy and physiology, to proceed to London and complete their clinical work in the larger field there open to them.

The advantages of studying at Cambridge are very great, and for those who can afford it there can be very little doubt that there are few, if any, more satisfactory ways of entering the medical profession at the present time than by joining a good college in Cambridge, after passing the Previous, and then at once commencing the study of the sciences which form the groundwork of medicine. After the examinations in anatomy and physiology have been passed it is easy to adjourn to London, and return for the degree when the necessary clinical studies have been completed.

Addenbrooke's Hospital, at which the clinical instruction is given in Cambridge, contains only 158 beds, but it does a great deal of good work, and offers quite as large a field as is necessary for the first two or three years, during which the student's time is of necessity almost entirely engaged in working at anatomy, physiology, and the allied sciences.

The fee for pupilship at the Addenbrooke's Hospital is eight guineas.

### University of Durham College of Medicine, Newcastle-upon-Tyne.

The Royal Infirmary, where the clinical instruction is given, contains 280 beds, but a large new hospital is being erected.

Residence at Newcastle for one year out of the five is necessary for all medical students desiring to graduate at the Durham University.

The composition fee for the college courses is 70 guineas, and that for the hospital practice 25 guineas, with small additional payments in certain cases.

### Owens College (Medical Department), Manchester.

This is one of the colleges of the Victoria University.

The Manchester Royal Infirmary, at which the medical instruction is given, contains 300 beds, and in connection with it there is a convalescent hospital (the Barnes Convalescent Hospital), containing 136 beds.

Instruction in mental diseases is given at the Royal Lunatic Asylum, and in infectious diseases at the Fever Hospital. Owens College, Manchester, of which the medical school is a department, is a large and most complete educational institution.

Large additions have lately been made to the buildings, so as to give the fullest possible opportunities for teaching in those departments more especially important to the students entering for medicine, namely, anatomy, physiology, pathology, and materia medica. But in every direction Owens College is well equipped for its work.

The composition fee for the college courses is £70, and for the hospital practice 40 guineas.

University College (Medical Faculty), Liverpool.

This, again, is one of the colleges of the Victoria University. It possesses some very fine and well-equipped laboratories, some of which have only recently been opened, and more are still building, a new medical department being at the present time in course of erection. Many very liberal gifts have been made to this institution, and everything is being carried out in a most complete manner, so that it has become a thoroughly organised and well-equipped medical school.

Numerous scholarships and prizes, amounting to over £600, are awarded annually.

In connection with this college is the now well-known Liverpool School of Tropical Medicine.

The composition fee for the college classes is £70, and that for the hospital practice is 40 guineas.

The Yorkshire College (Medical Department), Leeds.

This also is one of the colleges of the Victoria University.

The Leeds Infirmary, at which the clinical instruction is given, has more than 400 beds in constant use, and has attached to it a convalescent hospital. Mental diseases are studied at the West Riding Asylum, and the practice of the Leeds Public Dispensary, the City Fever Hospital and the Hospital for Women and Children is also open to the students. The new buildings for the Medical School were opened only four years ago, and they are very complete and well equipped.

The composition fee for the college courses is £67 4s., and that for the hospital practice 40 guineas.

The University of Birmingham (Faculty of Medicine).

To obtain a medical degree in the University of Birmingham it is necessary to have attended the classes of the University for at least the first four years of the curriculum.

The clinical instruction is given at the General Hospital and the Queen's Hospital, under the direction of the Clinical Board. These hospitals have a total of upwards of 400 beds, and are thoroughly well equipped for teaching purposes. The scientific teaching is given by the University professors and lecturers.

The composition fee for the lectures is 80 guineas, and that for the hospital practice 40 guineas.

University College (Faculty of Medicine), Bristol.

It is now arranged that students of the college shall be admitted to the clinical practice of the Bristol Royal Infirmary and the Bristol General Hospital conjointly. These institutions have between them a total of 470 beds, and complete departments for the various specialities, in addition to which they may attend the practice of certain special hospitals in town.

The composition fee for the lectures is 65 guineas, and that for the hospital practice 35 guineas.

University College (Department of Medicine), Sheffield.

The hospitals at which the clinical instruction is given are the Sheffield Royal Infirmary, containing 240 beds, the Sheffield Royal Hospital with 125 beds.

The composition fee for the lectures is 60 guineas, and for hospital practice £45.

University College, Cardiff, School of Medicine.

Three out of the five years of the curriculum may be spent at this school, the courses of study being recognised as qualifying for the Preliminary Scientific and Intermediate Examination in Medicine in the University of London, and for the first and second examination under the Conjoint Board.

Clinical instruction is given at the Cardiff Infirmary.

### SCOTTISH MEDICAL SCHOOLS.

Edinburgh University.

School of Medicine of the Royal Colleges, Edinburgh.

Medical College for Women, Edinburgh.

Glasgow University.

Queen Margaret College, Glasgow (the Women's Department of the University).

St. Mungo's College, Glasgow.

Anderson's Medical College, Glasgow.

University of Aberdeen.

University College, Dundee. Connected with the University of St. Andrews.

### IRISH MEDICAL SCHOOLS.

The School of Physic in Ireland, Dublin.

The Catholic University Medical School, Dublin.

The Schools of Surgery of the Royal Colleges of Surgeons in Ireland, Dublin.

Queen's College, Belfast.

Queen's College, Cork.

### MEDICAL SCHOOLS AND MEDICAL QUALIFICATIONS FOR WOMEN.

The following are the qualifying bodies which admit women as candidates for their degrees or diplomas, viz., the University of London, the University of Durham, the University of Edinburgh, the University of Glasgow, the University of St. Andrews, and the Royal University of Ireland; the Society of Apothecaries of London, the Conjoint Board of the Royal Colleges of Physicians and Surgeons in Scotland, and the similar Conjoint Board in Ireland.

The regulations of the General Medical Council in regard to medical education and examinations, apply to women exactly as they do to men.

In regard to place of study, women can obtain a complete course of medical education by attaching themselves to the London School of Medicine for Women; the Medical College for Women, Edinburgh; Queen Margaret College, Glasgow; the Schools of Surgery of the Royal College of Surgeons in Ireland; and the Queen's Colleges in Ireland situated in Belfast, Cork, and Galway.

It is worth remembering that the London and the Edinburgh Schools receive women only, and that at the Schools of Surgery of the Royal College of Surgeons in Ireland special rooms are set apart for women students.

They can also enter at the above-mentioned Universities, but they may not at all of them be able to obtain hospital practice. Ladies wishing to enter the medical profession cannot do better than apply to the Secretary of the London School of Medicine for Women, who will no doubt give them full information on the subject.

